# A Defined Antigen Skin Test for Diagnosis of Bovine Tuberculosis in Domestic Water Buffaloes (*Bubalus bubalis*)

**DOI:** 10.3389/fvets.2021.669898

**Published:** 2021-08-16

**Authors:** Tarun Kumar, Mahavir Singh, Babu Lal Jangir, Devan Arora, Sreenidhi Srinivasan, Devender Bidhan, Dipin Chander Yadav, Maroudam Veerasami, Douwe Bakker, Vivek Kapur, Naresh Jindal

**Affiliations:** ^1^College of Veterinary Sciences, Lala Lajpat Rai University of Veterinary and Animal Sciences, Hisar, India; ^2^Department of Veterinary Pathology, Lala Lajpat Rai University of Veterinary and Animal Sciences, Hisar, India; ^3^Haryana Pashu Vigyan Kendra, Lala Lajpat Rai University of Veterinary and Animal Sciences, Hisar, India; ^4^Huck Institutes of the Life Sciences, The Pennsylvania State University, University Park, PA, United States; ^5^Department of Livestock Production Management, Lala Lajpat Rai University of Veterinary and Animal Sciences, Hisar, India; ^6^Cisgen Biotech Discoveries Pvt. Ltd., Chennai, India; ^7^Independent Researcher, Lelystad, Netherlands; ^8^Department of Animal Science, The Pennsylvania State University, University Park, PA, United States; ^9^Department of Veterinary Public Health and Epidemiology, Lala Lajpat Rai University of Veterinary and Animal Sciences, Hisar, India

**Keywords:** water buffaloes (*Bubalus bubalis*), defined antigen skin test, sensitivity, specificity, bovine tuberculosis

## Abstract

Bovine tuberculosis (bTB) remains endemic in domestic water buffaloes (*Bubalus bubalis*) in India and elsewhere, with limited options for control other than testing and slaughter. The prescribed tuberculin skin tests with purified protein derivative (PPD) for diagnosis of bTB preclude the use of Bacille Calmette-Guérin (BCG)-based vaccination because of the antigenic cross-reactivity of vaccine strains with *Mycobacterium bovis* and related pathogenic members of the *M. tuberculosis* complex (MTBC). For the diagnosis of bTB in domestic water buffaloes, we here assessed a recently described defined-antigen skin test (DST) that comprises overlapping peptides representing the ESAT-6, CFP-10 and Rv3615c antigens, present in disease-causing members of the MTBC but missing in BCG strains. The performance characteristics of three doses (5, 10 or 20 μg/peptide) of the DST were assessed in natural tuberculin skin test reactor (*n* = 11) and non-reactor (*n* = 35) water buffaloes at an organized dairy farm in Hisar, India, and results were compared with the single intradermal skin test (SIT) using standard bovine tuberculin (PPD-B). The results showed a dose-dependent response of DST in natural reactor water buffaloes, although the SIT induced a significantly greater (*P* < 0.001) skin test response than the highest dose of DST used. However, using a cut-off of 2 mm or greater, the 5, 10, and 20 μg DST cocktail correctly classified eight, 10 and all 11 of the SIT-positive reactors, respectively, suggesting that the 20 μg DST cocktail has a diagnostic sensitivity (Se) of 1.0 (95% CI: 0.72–1.0) identical to that of the SIT. Importantly, none of the tested DST doses induced any measurable skin induration responses in the 35 SIT-negative animals, suggesting a specificity point estimate of 1.0 (95% CI: 0.9–1.0), also identical to that of the SIT and compares favorably with that of the comparative cervical test (Se = 0.85; 95% CI: 0.55–0.98). Overall, the results suggest that similar to tuberculin, the DST enables sensitive and specific diagnosis of bTB in water buffaloes. Future field trials to explore the utility of DST as a defined antigen replacement for tuberculin in routine surveillance programs and to enable BCG vaccination of water buffaloes are warranted.

## Introduction

Bovine tuberculosis (bTB) is a chronic inflammatory disease of cattle caused by members of the *Mycobacterium tuberculosis* complex, and in addition to being an important animal health problem, bTB also poses a significant threat to public health (1). It has been estimated that annual worldwide economic losses associated with bTB are ~USD 3 billion (2). The disease is well-controlled in high-income countries, however, bTB remains endemic in most low- and middle-income countries (LMICs), including India where bTB has significant impacts in terms of decreased productivity, increased mortality and zoonotic threat. While national control programs involving test-and-cull strategies have proven to be hugely successful in high-income countries, such approaches are often not feasible in LMICs for both social and economic reasons.

India has the largest livestock population in the world, including nearly 191 million cows and 109 million buffaloes (3). Livestock rearing is one of the most important activities in the rural areas of the country, and for many individuals, it is the only source of livelihood. Haryana, a state in northern India, has a large number (~4.3 million) of the Murrah breed of domestic water buffaloes (*Bubalus bubalis*). Indigenous buffaloes are important economically, contributing nearly 35% of the country's total milk (3). Although bTB has been well-studied in cattle generally, studies on this disease in buffalo are scarce, especially for high-producing breeds like Murrah.

A recent meta-analysis on bTB in India reported a pooled prevalence of bTB of 4.3% (95% CI: 2.7, 6.7) in buffaloes, calculated from a total of 29,037 animals tested between 1942 and 2016 (4). Without the implementation of any disease control program, this current level of endemicity is predicted to increase in the coming years, especially given the predicted intensification of dairy farming in India. Given that test-and-cull-based control programs are not implementable in India, a vaccine-based intervention strategy may be a promising solution. The Bacille Calmette-Guérin (BCG) vaccine was initially developed for control of human tuberculosis by Albert Calmette and Camille Guérin and was first used in humans in 1921 (5). While there is evidence supporting BCG-induced protection against bTB, the vaccine has not yet been licensed for use in cattle due to the presence of cross-reactive antigens that interfere with the specificity of the tuberculin skin tests recommended by the World Organisation for Animal Health (6, 7).

Recent developments in the field of bTB diagnostics have focused on fit-for-purpose tools that can reliably differentiate infected and (BCG) vaccinated animals (DIVA) in order to make implementation of BCG-based intervention strategies a possibility in LMICs. Several antigens with DIVA capability have been evaluated, of which ESAT-6, CFP-10, and Rv3615c appear to be the most promising (8, 9). These antigens have been extensively evaluated both in experimental and field conditions, and a peptide-based formulation of these antigens, henceforth referred to as the defined antigen skin test (DST), was previously evaluated for its utility in field studies in cross-bred cattle both in natural reactors and in BCG vaccinates (9, 10). Here, we assess the utility and performance of DST in buffaloes in India.

## Materials and Methods

### Antigens and Peptides

The DST used in the study is comprised of *M. bovis* antigens ESAT-6, CFP-10, and Rv3615c. Peptides (*n* = 13) representing these antigens were chemically synthesized at >98% purity by GenScript USA, Inc. and USV Private Limited, India (see Supplementary Table 1 for peptide sequences). The safety of DST has been demonstrated in *Bos taurus* subsp. *indicus* under Good Laboratory Practice (GLP) conditions in India with repeat and overdosing experiments (unpublished data). The bovine tuberculin (PPD-B) and avian tuberculin (PPD-A) were sourced from Prionics, Thermo Fisher, Schlieren, Switzerland.

### Animals

To determine the performance characteristics of DST in buffaloes, skin tests were conducted in adult female Murrah buffaloes (3–5 years old) recruited from an animal farm at the Lala Lajpat Rai University of Veterinary and Animal Science (LUVAS), Hisar, Haryana, India. Recruited animals that were known bTB reactors based on prior single intradermal testing with PPD-B (*n* = 11), were housed separately from the healthy herd. Skin tests were also conducted in control naïve animals (*n* = 35) from the organized dairy farm. All animal experiments were approved by the Institutional Animal Ethics Committee (IAEC) of the institute (letter number VCC/IAEC/2590-2619 dated 27-12-2018).

### Interferon-Gamma Enzyme-Linked Immunosorbent Assay

For *in vitro* stimulation of whole blood, PPD-B and PPD-A were used at a final concentration of 300 and 250 IU/ml, respectively, as per the BOVIGAM™ kit (Thermo Fisher Scientific) instructions. The DST peptide cocktail was used at 10 μg/ml in *in vitro* assays. Whole blood was collected and stimulated overnight at 37°C, 5% CO_2_, with the antigens *in vitro*. BOVIGAM™ kits were used to determine IFN-γ concentrations in whole-blood culture supernatants. Results for antigen-stimulated cultures were expressed as background-corrected optical density at 450 nm (i.e., ΔOD450).

### Intradermal Skin Test Procedures

Skin tests using PPD-tuberculins (PPD-A at 25,000 IU/ml and PPD-B at 30,000 IU/ml) were performed as recommended by the manufacturer (Thermo Fisher Scientific, USA), and the results were interpreted as per OIE guidelines (11). Skin thickness was measured by the same operator before administration of PPD and at 72 h post-injection. Skin test readings were measured in millimeters as per OIE-prescribed guidelines. The DST was administered in three different doses, i.e., 5, 10, and 20 μg of each peptide constituent (final injection volume = 0.1 ml), and the sites of injection were randomized. For the single intradermal test (SIT) involving only PPD-B, an increase in skin thickness of 4 mm or more was considered a positive reaction (11), whereas for DST, an increase in skin thickness of 2 mm or more was considered a positive reaction based on the studies conducted in cattle (9). For the comparative cervical test (CCT) involving injection of both PPD-A and PPD-B, a difference in increase in skin thickness between the two infection sites (B-A) is calculated. Per OIE guidelines, a measurement of >4 mm was considered positive for CCT.

### Statistical Analyses

All statistical analyses were performed using Prism 8 (GraphPad Software, La Jolla, CA). Confidence intervals (CI) for the sensitivity estimates in natural reactors for DST and PPDs were calculated using the Clopper-Pearson method. For PPD-B, we calculated a one-sided CI (lower 95% CI: 76). Standard two-sided CIs were calculated for CCT, i.e., PPD (B-A) and DST using the same method with point estimates of 82% (95% CI: 48, 98).

## Results

### Performance of the Defined Antigen Cocktail in the *in vitro* IFN-γ Release Assay

**Figure 1:**
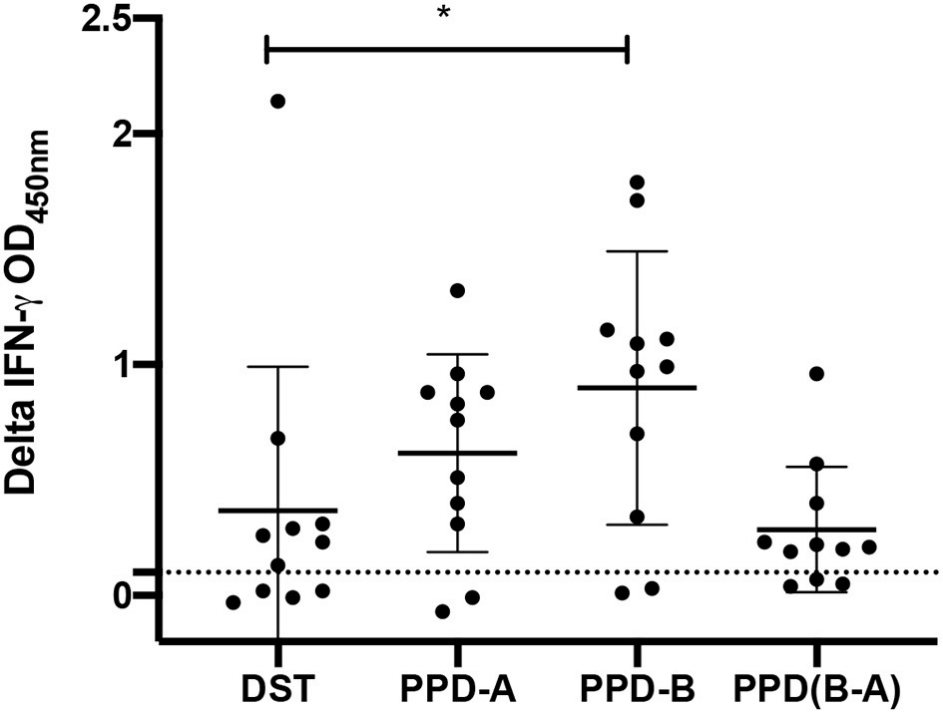
Capacity of DST antigens to induce an *in vitro* IFN-γ response in whole blood collected from naturally infected buffaloes (*n* = 11). The background-corrected optical density (OD) values for all antigens tested are shown. The horizontal line indicates the mean (±SD), and the statistical difference between the responses was determined by using the paired *t*-test (*P < 0.05). The means of background-subtracted OD values for DST, PPD-A, PPD-B, and PPD(B-A) were found to be 0.37, 0.62, 0.90, and 0.29, respectively. The standard deviations for DST, PPD-A, PPD-B, and PPD(B-A) were 0.62, 0.43, 0.60, and 0.27, respectively.

IGRAs were conducted to compare the performance of the DST peptide cocktail, composed of ESAT-6, CFP-10, and Rv3615c, with that of the PPDs (Figure 1). The data demonstrated that PPD-B induced a significantly greater IFN-γ response in reactor animals when compared to the DST cocktail (*P* < 0.05; mean of difference = 0.053), while there was no statistical significance in the response induced by DST vs. PPD-B minus PPD-A (*P* = 0.98). Out of 11 animals tested, nine showed IGRA positivity (IGRA cut-off > 0.1) when PPD-B alone was considered, eight were IGRA-positive by PPD-B minus PPD-A, and seven were IGRA-positive by DST; however, the differences were not statistically significant. The data suggest that the DST peptide cocktail may be used as a stimulating antigen in blood tests for diagnosis of bTB in buffaloes.

### Defined Antigens Induce a Sensitive and Specific Skin Test Response

**Figure 2:**
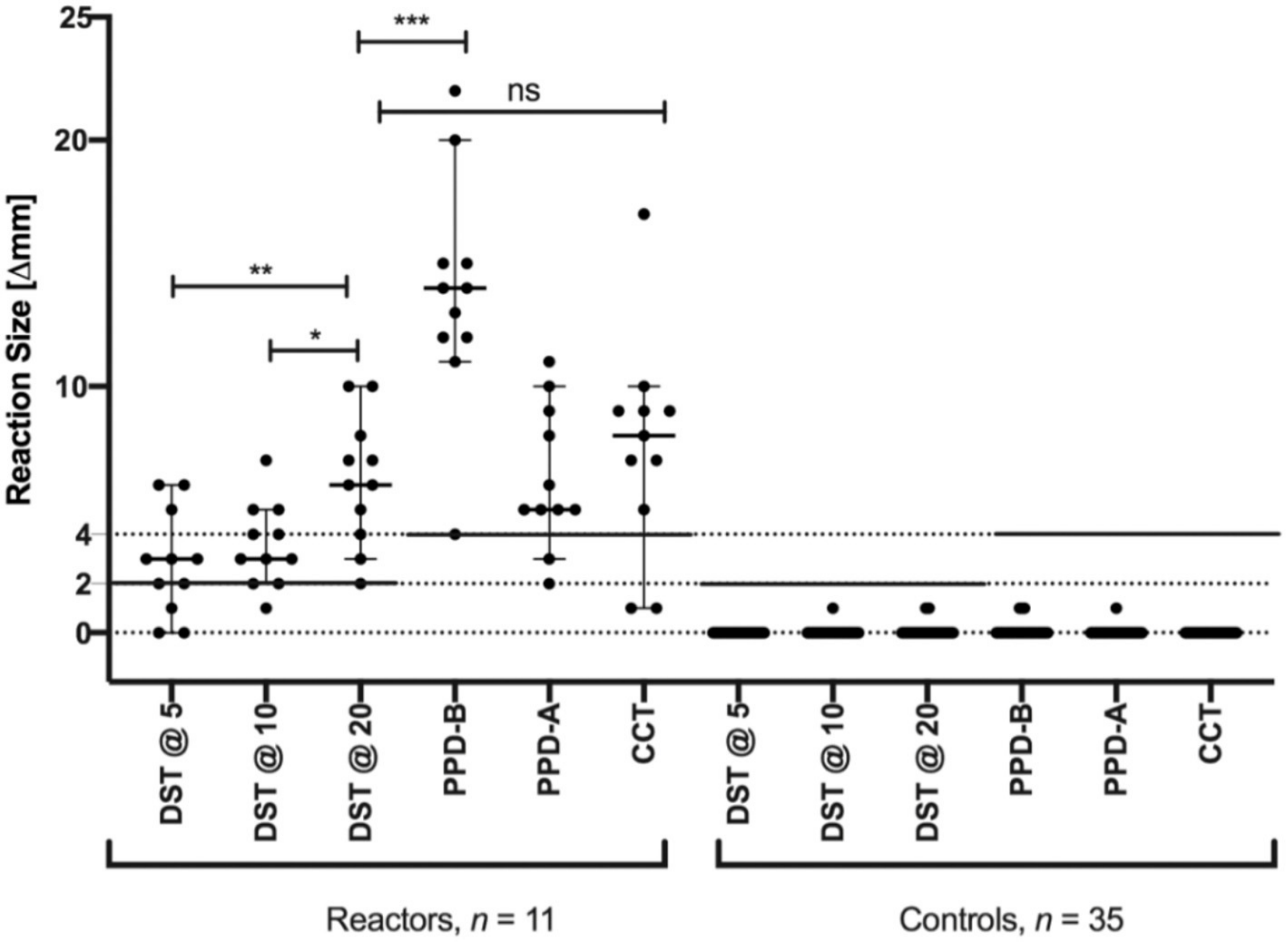
Skin test responses for PPD-A, PPD-B and DST at three doses (5, 10, and 20 ug/ml). Responses were measured at 72 h after injection in naturally infected (*n* = 11) and naive control (*n* = 35) buffaloes. Results are expressed as the difference in skin thickness (in millimeters) between the pre- and post-skin-test readings, with the horizontal line providing the median (±95% CI). The statistical difference between the responses was determined using analysis of variance (ANOVA) (*P < 0.05; **P < 0.01; ***P < 0.001). The solid horizontal lines at 2 and 4 mm are the cut-offs used for DST, and CCT and PPD-B, respectively. The mean skin thickness recorded for DST@5, DST@10, DST@20, PPD-B, PPD-A, and CCT in reactor buffaloes are 2.8, 3.5, 6.2, 13.8, 6.3, and 7.5 mm respectively.

The performance of the DST was assessed in natural reactor (*n* = 11) and non-reactor (*n* = 35) buffaloes. The results showed that, when using a cut-off of 2 mm or more, the DST cocktail at 5, 10, and 20 μg correctly classified eight, 10 and 11 of the 11 reactors as positive, respectively (Figure 2 and Table 1). The standard bovine tuberculin antigen (PPD-B), used in the SIT, induced a significantly stronger skin test response than that induced by the highest dose of DST used in this study (*P* < 0.001) and identified all 11 reactors as positive, whereas the CCT identified nine of the 11 buffaloes as reactors. Notably, none of the tested DST doses induced any measurable skin induration responses in the control group (*n* = 35).

**Table 1:** Relative sensitivities of SIT, CCT, and DST at different doses.

**Test^**a**^**	**Positive**	**Negative**	**Sensitivity**
SIT^b^	11	0	100% (lower 95% CI: 71.5)
CCT	9	2	84.6% (95% CI: 54.5, 98.1)
DST, 5 μg	8	3	78.6% (95% CI: 49.2, 95.3)
DST, 10 μg	10	1	91.7% (95% CI: 61.5, 99.8)
DST, 20 μg^b^	11	0	100% (lower 95% CI: 71.5)

a *The diagnostic specificity for all tests listed was found to be 100% (lower 95% CI: 71.5)*.

b *The SIT and the DST with 20 μg identified all 11 natural reactors as test-positive*.

## Discussion

Recent developments in the field of bTB diagnostics have centered around identifying candidate antigens for DIVA in order to enable vaccine-based interventions in LMICs. Previous studies on the use of defined skin test antigens in cattle, using a combination of ESAT-6, CFP-10, MPB70, and MPB83, concluded that these antigens are promising candidates for performing the IGRA in cattle, with the ability to differentiate between vaccinated animals and those infected with *M. bovis* (8, 12). The current study was performed to assess the performance of a recently developed peptide-based DST cocktail in Murrah buffaloes, an economically important breed of buffaloes in India. In this study, we first performed IGRAs with DST peptides at 10 μg/ml. Although PPD-B was the most sensitive in eliciting IFN-γ responses *in vitro*, there was no significant difference in the responses elicited by the DST peptides and PPD(B-A). To our knowledge, this is the first study where potential of defined skin test antigens has been demonstrated in naturally tuberculin skin test reactor domestic buffaloes. Our data demonstrated that, these peptide-based antigens have promising potential for use in IGRAs as an ancillary test in concert with the skin test for distinguishing between reactor and uninfected animals. However, as this proof-of-concept study has only been performed on a small number of animals, it will be important to validate the performance of DST in a larger cohort of known infected and naïve buffaloes.

We also assessed the utility of the DST peptide cocktail as a skin test reagent in buffaloes. Of the three different doses (5, 10, and 20 μg per peptide) of DST that were compared, 20 μg/ml appeared to have the highest sensitivity, although higher concentrations may need to be tested to achieve an optimal balance between sensitivity and specificity. Most importantly, the peptide cocktail also proved to be highly specific, as no measurable skin induration response was observed following injection into naïve uninfected buffaloes. Both SIT and the highest dose of DST were found to be 100% sensitive, identifying all 11 naturally-infected buffaloes as reactors. In this context, it is important to note that, given the high burden of environmental mycobacteria in India, PPD-B can induce non-specific responses, often leading to high rates of false-positivity with SIT. The CCT with the simultaneous injection of PPD-A helps overcome this loss of specificity associated with SIT, but only at the expense of sensitivity. The results of this study showed that the peptide-based DST provides sensitivity and specificity equivalent to that of the OIE-recommended CCT, without the need for a second injection. Moreover, DST at its highest tested dose (20 μg/ml) elicits a lower amplitude of skin test response in infected animals (6.18 ± 2.60 mm) as compared with PPD-B (13.81 ± 4.68 mm) without hindering the specificity estimate. Such exuberant non-specific PPD-B responses tend to raise concerns and limit acceptability of the SIT among farmers. In reactor buffaloes, we found that 20 μg DST produced a skin response greater than the cut-off of 2 mm in all of the animals tested. In this context, it is crucial to highlight the difference in antigen dosage that may be necessary for accurate bTB diagnosis in cattle and buffaloes. In a previous study with cross-bred cattle, pilot dose titration experiments showed that DST@10 may be the optimal dose in cross-bred cattle (9). However, here the data show that DST@20 may be better suited for bTB diagnosis in buffaloes as it identified all reactors as test-positive without compromising test Specificity. Future studies that are adequately statistically powered are required to accurately determine the antigen dosage required for diagnosis in buffaloes.

In 2019, the World Health Organization reported that over nine million people developed TB, of which ~1.5 million died (13). Only eight countries account for about two-thirds of the total reported cases of TB, with India leading the count. Moreover, some recent estimates suggest that ~9% of all human TB cases in India may be of zoonotic origin (14). Hence, it is increasingly recognized that controlling TB in the livestock population in these settings could be a major step toward attaining the ambitious End TB goal of 2035 (15). As part of these control efforts, effective methods of identifying infected animals are needed. However, there are several major limitations to the OIE-recommended tuberculin-based skin tests, including the fact that the active components in the reagent are undefined and their interference with BCG-based vaccination. In contrast, the peptide-based DST offers superior quality control and potential for DIVA has previously been demonstrated in cattle (10), and the current study indicates that the DST antigens are promising candidates for differentiating between reactor and uninfected buffaloes. Limitations of the study include the relatively small number of animals and their origin from a single dairy farm. Hence, these results will need to be verified in larger cohorts of naturally infected and naïve animals to establish more refined estimates of sensitivity and specificity of the DST in buffaloes prior to consideration for replacement of the traditional tuberculin standard. Moreover, while the DIVA capability of DST enables implementation of BCG vaccination as an intervention, the efficacy of BCG vaccination in buffaloes remains unknown. Field trials are in process to evaluate the performance of DST in larger cohorts of infected, BCG-vaccinated and naïve buffaloes, and the efficacy of BCG in buffaloes in natural transmission settings in India is being planned.

## Data Availability Statement

The original contributions presented in the study are included in the article/Supplementary Material, further inquiries can be directed to the corresponding author/s.

## Ethics Statement

The animal study was reviewed and approved by Institutional Animal Ethics Committee, Lala Lajpat Rai University of Veterinary and Animal Sciences (LUVAS), Hisar, Haryana, India.

## Author Contributions

SS, VK, MV, DBa, and NJ conceptualized the study. TK, MS, BJ, DA, DBi, and DY conducted the animal experiments. MS, SS, and MV conducted the lab experiments. TK, MS, and SS analyzed the data. TK prepared the first draft. All authors contributed to the article and approved the submitted version.

## Conflict of Interest

MV was employed by company Cisgen Biotech Discoveries Pvt. Ltd. SS and VK have filed intellectual property protection for the DST. The remaining authors declare that the research was conducted in the absence of any commercial or financial relationships that could be construed as a potential conflict of interest.

## Publisher's Note

All claims expressed in this article are solely those of the authors and do not necessarily represent those of their affiliated organizations, or those of the publisher, the editors and the reviewers. Any product that may be evaluated in this article, or claim that may be made by its manufacturer, is not guaranteed or endorsed by the publisher.
